# Integrin alpha-V is an important driver in pancreatic adenocarcinoma progression

**DOI:** 10.1186/s13046-021-01946-2

**Published:** 2021-06-26

**Authors:** Marius Kemper, Alina Schiecke, Hanna Maar, Sergey Nikulin, Andrey Poloznikov, Vladimir Galatenko, Michael Tachezy, Florian Gebauer, Tobias Lange, Kristoffer Riecken, Alexander Tonevitsky, Achim Aigner, Jakob Izbicki, Udo Schumacher, Daniel Wicklein

**Affiliations:** 1 Department of General, Visceral and Thoracic Surgery, University Medical Centre Hamburg-Eppendorf, Martinistrasse 52, 20246 Hamburg, Germany; 2 Institute of Anatomy and Experimental Morphology, University Medical-Center Hamburg-Eppendorf, Hamburg, Germany; 3 Dmitry Rogachev Federal Research Center of Pediatric Hematology, Oncology and Immunology, Moscow, Russia; 4 Faculty of Mechanics and Mathematics, Lomonosov Moscow State University, Moscow, Russia; 5 Department of General, Visceral and Tumor Surgery, University Hospital Cologne, Köln, Germany; 6 Research Department Cell and Gene Therapy, Department of Stem Cell Transplantation, University Medical Centre Hamburg-Eppendorf, Hamburg, Germany; 7 Faculty of Biology and Biotechnology, Higher School of Economics University, Moscow, Russia; 8 Rudolf-Boehm-Institute for Pharmacology and Toxicology, Clinical Pharmacology, Medical Faculty, University of Leipzig, Leipzig, Germany

**Keywords:** Pancreatic cancer, Integrin alpha-V, Metastatic cascade, TGF-beta signaling, EMT

## Abstract

**Background:**

Mesothelial E- and P-selectins substantially mediate the intraperitoneal spread of Pancreatic ductal adenocarcinoma (PDA) cells in xenograft models. In the absence of selectins in the host, the integrin subunit alpha-V (ITGAV, CD51) was upregulated in the remaining metastatic deposits. Here we present the first experimental study to investigate if ITGAV plays a functional role in PDA tumor growth and progression with a particular focus on intraperitoneal carcinomatosis.

**Methods:**

Knockdown of ITGAV was generated using an RNA interference-mediated approach in two PDA cell lines. Tumor growth, intraperitoneal and distant metastasis were analyzed in a xenograft model. Cell lines were characterized in vitro. Gene expression of the xenograft tumors was analyzed. Patient samples were histologically classified and associations to survival were evaluated.

**Results:**

The knockdown of ITGAV in PDA cells strongly reduces primary tumor growth, peritoneal carcinomatosis and spontaneous pulmonary metastasis. ITGAV activates latent TGF-β and thereby drives epithelial-mesenchymal transition. Combined depletion of ITGAV on the tumor cells and E- and P-selectins in the tumor-host synergistically almost abolishes intraperitoneal spread. Accordingly, high expression of ITGAV in PDA cells was associated with reduced survival in patients.

**Conclusion:**

Combined depletion of ITGAV in PDA cells and E- and P-selectins in host mice massively suppresses intraperitoneal carcinomatosis of PDA cells xenografted into immunodeficient mice, confirming the hypothesis of a partly redundant adhesion cascade of metastasizing cancer cells. Our data strongly encourage developing novel therapeutic approaches for the combined targeting of E- and P-selectins and ITGAV in PDA.

**Supplementary Information:**

The online version contains supplementary material available at 10.1186/s13046-021-01946-2.

## Background

Pancreatic ductal adenocarcinoma (PDA) belongs to the most lethal malignancies in industrialized countries. By 2040 an increase to 777,423 death per year worldwide is expected [1]. Despite advances in diagnostics and therapy, the five-year survival rate is only 9% [1]. These data indicate that the vast majority of patients relapse within 5 years after surgery. Besides locoregional relapse or distant metastasis to lungs or liver, recurrence manifests as peritoneal carcinomatosis in many cases [2], making this process an important target for therapeutic intervention. In fact, the median overall survival for patients diagnosed with intraperitoneal carcinomatosis amounts 14.1 months only [2].

In a previous study, we demonstrated that tumor cell adherence to the peritoneal mesothelium is highly dependent on the binding of carbohydrate structures present of the cancer cells’ outer cell membrane to E- and P-selectins expressed on the peritoneal mesothelial cells using a xenograft model with selectin-deficient mice [3]. In the absence of selectins, only a minority of cells could adhere to the peritoneum, which considerably inhibited intraperitoneal tumor growth [3]. In the present study, we investigated the underlying mechanism for the remaining metastatic deposit in selectin-deficient mice in which we found Integrin αV (ITGAV) to be functionally involved. ITGAV has recently been reported to be an important driver of cancer progression in prostate [4, 5] and colon carcinoma [6]. In PDA, recent studies demonstrate that the dimer of Integrins αVβ6 (ITGAV / ITGB6) is overexpressed in most tumors (with overexpression being retained in the corresponding metastases) and is a feasible target for novel therapeutic approaches [7, 8].

## Methods

### Cell lines and RNA interference-mediated ITGAV knockdown

Authenticated PDA cell lines PaCa 5061 [9] and BxPC3 [10] were used. ITGAV knockdown was achieved using a short hairpin RNA (shRNA) mediated approach: A 65 bp hairpin DNA oligomer containing a 19 bp anti-ITGAV sequence (GGATGGTGTCCACTTCAAA) was inserted into the pLVX vector (Clontech). Potential off-target effects were checked (NCBI BLAST). An shRNA sequence against firefly luciferase was used for the control cell line [11]. Knockdown and control cells were seeded at three cells per well. Sublines with normal ITGAV and low ITGAV expression were pooled respectively and tested for Mycoplasma. The passage number of used cell lines never exceeded 20.

### Flow cytometry

Flow cytometry was performed as described [12]. Following antibodies were used: ITGAV (327,907, BioLegend), ITGB1 (11–0299, Thermo Fisher Scientific), ITGB3 (336,403, BioLegend), ITGB5 (11–0497-42, Thermo Fisher Scientific), ITGB6 (FAB4155P, R&D), HLA-DR (347,401, BD). Stained cells were subjected to FACS Calibur Flow Cytometry System (BD).

### Enzyme-linked immunosorbent assay (ELISA)

Cells were seeded in a T25 cell culture flask. After 48 h, they were changed to 1.5 mL serum-free medium. After another 24 h of incubation, the medium was harvested and centrifuged. Supernatants were collected. The measurement of TGF-β1 was made using the Free Active TGF-β1 ELISA Kit (cat. 437,707, BioLegend), respectively Total TGF-β1 ELISA Kit (cat. 436,707, BioLegend).

### In vitro characterization of ITGAV knockdown cells

Cell proliferation was assessed using the XTT assay (Roche Diagnostics). 3 × 10^3^ cells were plated per well (96-well plate) and incubated for 72 h. Fifty microliter XTT labeling mixture was added per well. After 5 h incubation, spectrophotometrical absorbance (450 nm) was measured using a microplate reader (MR5000 Multiplate Reader, Dynatech).

Static cell adhesion assays were performed in fibronectin-coated μ-Slides (ibidi). Cells were expanded to a concentration of 1 × 10^5^ cells and added to a suspension volume of 60 μL. After 1 h of incubation, visual fields were documented (Axio Cam MRm, Carl Zeiss). To calculate the fraction of adherent cells, slides were washed with PBS and the same predefined visual fields were documented.

Differences in cell migration were assessed using the FluoroBlok Migration Assay with a 24-well plate with 8.0-μm pore size inserts (BD Bioscience). Cells were trypsinized and resuspended in serum-free medium in a concentration of 3 × 10^5^ cells/ml. Four hundred microliter cell suspension was added to the apical chamber and 1200 μl medium with 10% FCS (Gibco) as chemoattractant was added to the bottom chamber. The assay was incubated for 24 h. After removing the chemoattractant from the bottom chamber, visualization of migrated cells was performed by adding 500 μl/well HBSS buffer with Calcein AM (Invitrogen) 4 μg/mL in the bottom well and incubating for 1 h. The readout was conducted at 485/530 nm (Ex/Em) on a Genios bottom-reading fluorescence plate reader (Tecan). To assess the invasive potential, cells were seeded on a 24-multiwell insert plate uniformly coated with basement membrane equivalent Matrigel (FluoroBlok Invasion System, BD Biosciences). After rehydration according to the manufacturer’s guidelines for use, all further steps were identical to those described for the migration assay.

For the colony-forming assay, 1500 μl single-cell suspension (1200 cells/ml) was mixed with 1500 μl growth factor reduced Matrigel (cat.: 356230, BD Biosciences). Fifty microliter of this suspension was seeded per well (96-well plate). After 30 min incubation, 200 μl medium was added per well. The plates were kept in an incubator for 14 d.

### Animal experiments: xenograft mouse model

C57BL/6 pfp^−/−^/rag2^−/−^ mice and E- and P-selectin double deficient pfp^−/−^/rag2^−/−^ [3] at the age of 8–12 weeks and bodyweight of 21–29 g were used for the study. For the intraperitoneal and the subcutaneous xenograft model, 1 × 10^6^ viable, mycoplasma-free tumor cells suspended in 200 μl RPMI were injected intraperitoneally or subcutaneously. Peritoneal carcinomatoses were quantified by an adapted peritoneal carcinomatosis index (PCI). Briefly, the murine peritoneum was divided into 9 sections (upper left to lower right) and every section was attributed with a carcinoma score from 0 to 3, resulting in a total PCI score of 0 to 27 for each animal [3, 13]. For the subcutaneous experiment, mice were injected subcutaneously directly under the right scapula with 1 × 10^6^ mycoplasma-free cells. Animals were sacrificed when primary tumors exceeded 1.5 cm^3^ or ulcerated the mouse skin. All animal experiments were performed following the United Kingdom Coordinating Committee of Cancer Research Guidelines [14]. The experiment was approved by the local licensing authority (project no. G09/88 and G10/55).

### Quantitative real-time polymerase chain reaction (qRT-PCR)

After sacrifice, the left lungs were homogenized (TissueLyser II, Qiagen) and subjected to DNA isolation (QIAamp DNA Mini Kit, Qiagen). Two hundred microliter blood was subjected to DNA isolation. DNA concentrations were quantified (NanoDrop, Peqlab). All lung DNA samples were normalized to 30 ng/μL. The concentrations of blood DNA were similar in all samples (approx. 10 ng/μL) and were therefore not normalized. qRT-PCR was performed with established human-specific Alu primers [15]. Numerical data were determined against a standard curve as described [16].

### Western blot

Protein concentration was determined by bicinchoninic acid assay. Western blots were performed as described [17]. Briefly, 30 μg of protein of each sample was added per well. Electrophoresis was performed in 10% polyacrylamide separating gel with a 5% stacking gel. Proteins were transferred to nitrocellulose membranes (0.45 μm, GE Healthcare). After overnight incubation in blocking solution (5% milk powder), membranes were incubated with the respective antibody: ITGAV (cat. sc-376,156, Santa Cruz), ITGB1 (cat sc-374,429, Santa Cruz), ITGB6 (cat. c-293,194, Santa Cruz) and a c-terminal SMAD 4 antibody (cat. ab217267, Abcam). HSC70 (cat. Sc-7298, Santa Cruz) was used as intrinsic control. Goat anti-Mouse IgG (cat. P0447, Dako) or, respectively, Goat anti-rabbit IgG (cat. Sc-2054, Santa Cruz) were used as a secondary antibodies.

### Affymetrix gene arrays

RNA was extracted (miRNeasy Mini Kit, Qiagen) and concentrations were determined (NanoDrop). The RNA integrity number was higher than 7 for all samples (Agilent Bioanalyzer 2100 system). Expression analysis was performed using the GeneChip Human Transcriptome Array 2.0 (Thermo Fisher Scientific). For cDNA synthesis, labeling and hybridization, the GeneChip WT PLUS Reagent Kit and GeneChip Hybridization, Wash, and Stain Kit (Thermo Fisher Scientific) were used.

### Immunohistochemical analysis

Sections of formalin-fixed, paraffin-embedded tumors were used. Briefly, after antigen retrieval, the following primary antibodies were used: ITGAV (sc-376,156, Santa Cruz,); ITGB1 (AB3167, Abcam), ITGB6 (HPA023626, Atlas), CEACAM7 (LS-B13068, LSBio), TGFBR1 (PA5–98192, Thermo Scientific), phospho-SMAD2 (AB3849, Millipore), TGFBI (MA526731, Thermo Fisher Scientific), Twist (ab50581, Abcam), Ki 67 (M7240, Agilent); human-specific fibronectin (NCL-FIB, Leica Biosystems); fibronectin, cross-reacting with the fibronectin-equivalent protein in mouse (A0245, Dako); STAT1 (HPA000931, Sigma-Aldrich), HLA-DR (M0746, Agilent). Vectastain ABC kit (Vector) was used. Slides were scanned by Axio Scan Z1 (Zeiss). For quantification with Image J (NIH), images of four randomly chosen vision fields (not containing necrotic areas) were analyzed. If artifacts interfered with automated quantification by Image J, the staining was quantified by two experienced investigators: grade 0: no reaction or weak focal reaction; grade 1 intense focal or diffuse weak reaction; grade 2 moderate diffuse reaction; grade 3 for an intense diffuse reaction.

### Study population

Two hundred nine patients with PDA who underwent pancreaticoduodenectomy at the University Medical Centre Hamburg-Eppendorf between 2000 and 2012 were included. Written informed consent was obtained from all participants. The study protocol was approved by the Hamburg Medical Chamber’s ethics committee (Approval number: PV3548).

### Tissue microarray (TMA) construction and analysis

TMA construction was performed as previously described [18]. Only punches with clearly detectable tumor tissue were included. The staining intensity and quantity of the tumor cells of each tissue spot were scored. To analyze the phospho-SMAD2 staining, all cell nuclei in a visual field were counted and the percentage of phospho-SMAD2 -positive cell nuclei was determined. If this was more than 75%, the tissue sample was classified as high. Of the initial 209 samples, ITGAV could be analyzed in 183, pSMAD2 in 180, STAT1 in 160, and HLA-DR in 192 samples. Immunohistochemical analysis of the sections was performed without knowledge of the patients’ identity or clinical status.

### Data analyses

GraphPad Prism was used for in vitro and in vivo statistical calculations (Student’s t-test, Kaplan-Meier method, Log-rank test). To analyze the TMA, SPSS was used: Relationships between categorical variables were calculated using chi-square tests. Survival curves were plotted using the Kaplan-Meier method and analyzed by the log-rank test. For data obtained by gene expression array, statistical analysis was performed using Transcriptome Analysis Console (4.0.0.25) with summation method Gene + Exon - SST-RMA (version 1) and empirical Bayes method. Calculated *p*-values were corrected for multiple testing using the Benjamini-Hochberg (FDR) procedure. The sample sets with expression fold changes of at least +/− 1.5 and with an FDR *p-*value < 0.05 were considered significantly differentially expressed. Further analysis was performed with R 3.5.1 programming language with IDE RStudio 1.1. The heat maps were constructed with the “heatmap.2” function from “gplots” package.

## Results

### Integrin expression was upregulated in intraperitoneal carcinomatosis grown in selectin-deficient mice

**Fig. 1:**
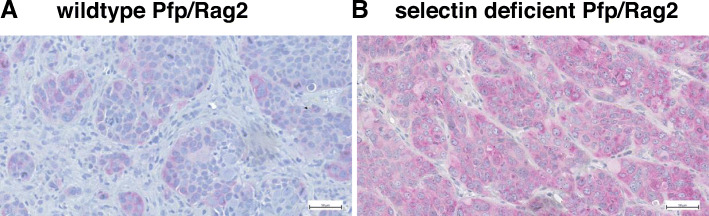
In a xenograft model of human PDAC, Integrin αV is upregulated in intraperitoneal carcinomatoses grown in E- and P-selectin knockout mice. Immunohistochemical staining for human Integrin αV: Carcinomatosis from wild-type (**a**) compared with those grown in E−/P-selectin-deficient pfp^−−^/rag2^−−^ mice (**b**). Scale bar: 50 μm

**Fig. 2:**
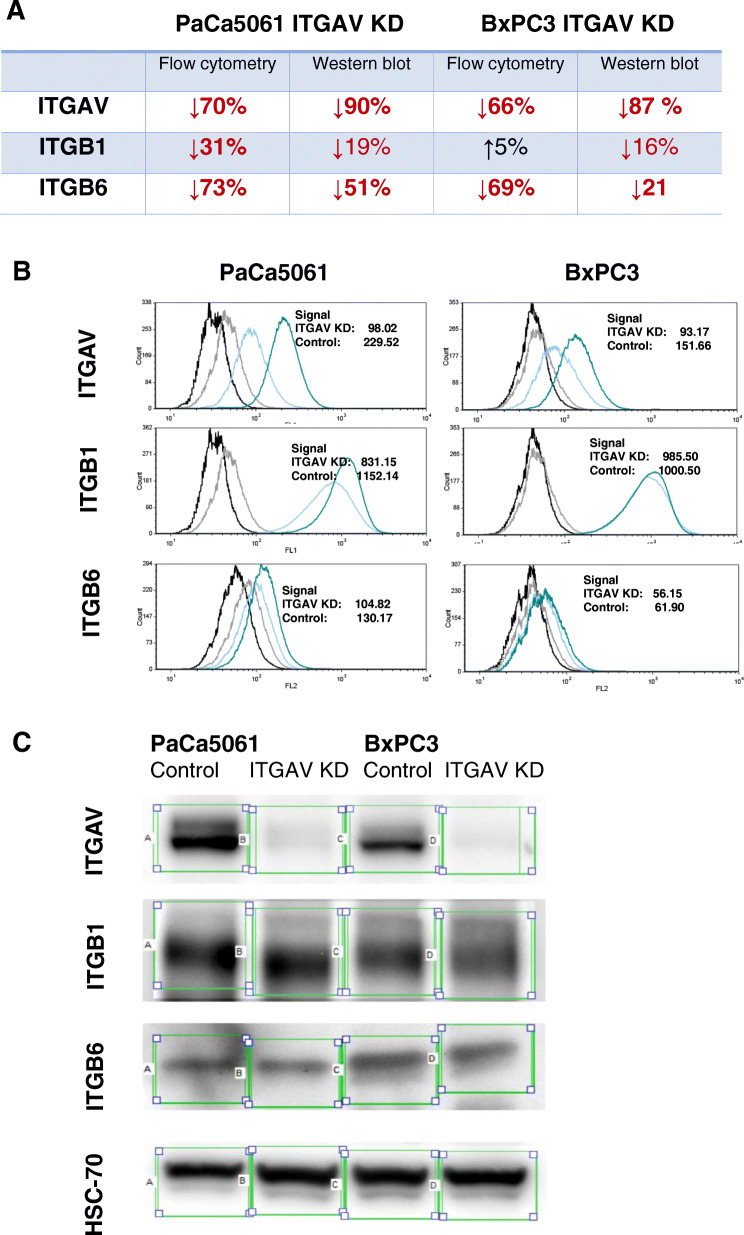
Changes of ITGAV and corresponding beta-subunits ITGB1 und ITGB6 (**a**) using flow cytometry (**b**) and Western blot (**c**). The expression of ITGAV was reduced by > 65% in PaCa 5061 and BxPC3 ITGAV KD cells. Subunits ITGB1 and ITGB6 were found to be downregulated on PaCa 5061 ITGAV KD cells*.* In contrast, only ITGB6 was downregulated on BxPC3 ITGAV KD cells, while the expression of ITGB1 remained almost unchanged

The gene expression profiles of tumors that had developed in the host animals despite selectin deficiency were compared with the gene expression profiles in tumors grown in wild-type *Pfp*^*−/−*^/*Rag2*^*−/−*^ mice: Integrins β1 (1.56-fold), α2 (1.67-fold), β6 (1.69-fold) and αV (1.70-fold) were found to be upregulated in the tumors of the selectin deficient animals. We selected integrin alpha-V (ITGAV) - which displayed the highest upregulation - to further study integrin influence in PDA. We confirmed the upregulation of ITGAV in xenograft carcinomatoses using immunohistochemistry (Fig. 1). We then generated a subline of the PaCa 5061 cell line with stable knockdown of ITGAV (shRNA), termed PaCa 5061 ITGAV KD and a respective control subline with unchanged ITGAV designated as PaCa 5061 control. The expression of ITGAV was reduced by > 65% in PaCa 5061 as determined by flow cytometry and western blot (Fig. 2a).

### ITGAV KD massively suppressed intraperitoneal carcinomatosis and reduced primary tumor development and distant metastasis in SMAD4-intact PaCa 5061 cells

**Fig. 3:**
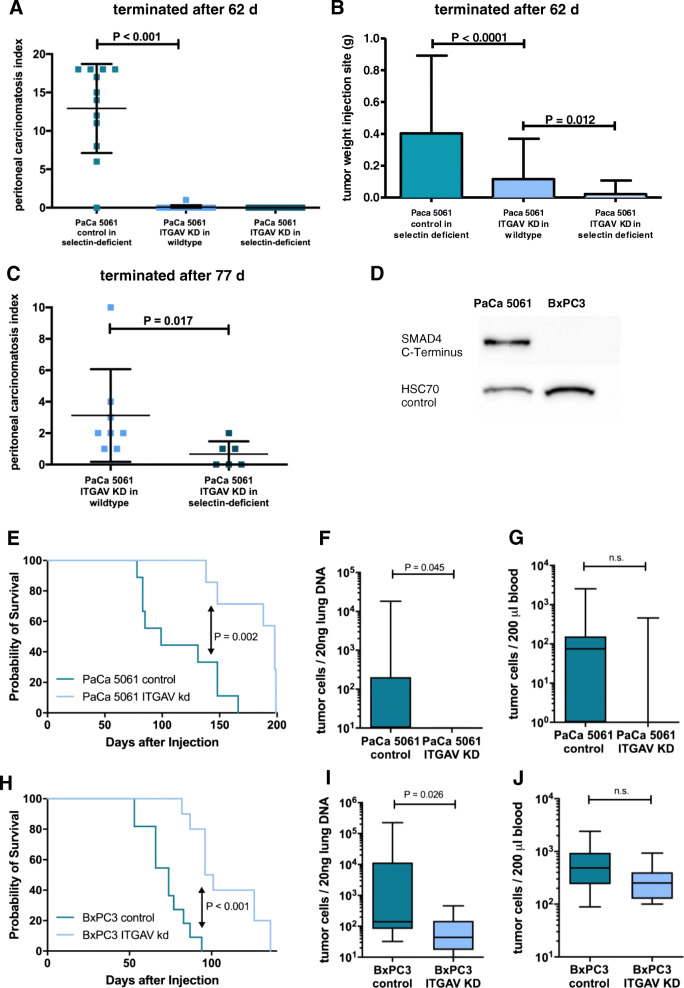
Knockdown of ITGAV in PaCa 5061 led to an effective reduction of intraperitoneal carcinomatosis after 62 d compared with control PaCa 5061 in E−/P-selectin deficient mice: Only 1/15 animals of the PaCa 5061 ITGAV KD group in wild-type mice showed carcinomatosis formation vs. 11/12 mice with carcinomatoses in the control group (*P* <  0.0001, **a**). ITGAV knockdown also reduced tumor growth at the injection site (*P* <  0.0001, B). 7/15 (ITGAV KD cells in E−/P-selectin k.o. mice) displayed tumors at the injection site vs. 11/12 in the control group (control PaCa 5061 in E−/P-selectin deficient mice). Combination of ITGAV KD and selectin knockout led to further reduction of injection site tumors (*P* = 0.012; only 2/14 animals with tumors, **b**). After 77 days, this synergistic effect of ITGAV and E−/P-selectins could also be demonstrated for intraperitoneal carcinomatosis formation (*P* = 0.017, C; 8/13 mice with tumor take with all eight displaying carcinomatosis with ITGAV KD in wild-type mice vs. 6/14 mice with tumor take and 3 of them displaying carcinomatosis with ITGAV KD in E−/P-selectin deficient animals). Using western blot, the C-terminal deletion of SMAD 4 in BxPC3 cells was confirmed (**d**). Wild-type mice inoculated subcutaneously with PaCa 5061 ITGAV KD cells showed significantly prolonged survival (tumor take rate 7/10) compared with control cells in wild-type animals (take rate 9/10; *P* = 0.002, **e**). Similar results were found for BxPC3 (100% take rate, both groups; *P* <  0.001, 2H). The number of human cells in the animals’ lungs was significantly reduced in mice injected with PaCa 5061 (*P* = 0.045, **f**) and BxPC3 ITGAV KD cells (*P* = 0.045, I). There was no significant difference for the PaCa5061 (**g**) or BxPC3 tumor cells circulating in the animals’ blood (**j**)

To answer the question of whether ITGAV on the tumor cells alone or in combination with E- and P-selectins in the tumor environment has a functional effect on intraperitoneal carcinomatosis formation, PaCa 5061 ITGAV KD and PaCa 5061 control cells were intraperitoneally injected into selectin-deficient and wild-type *Pfp*^*−/−*^/*Rag2*^*−/−*^ mice (12 animals each group for the selectin-deficient mice, 14 and 15 selectin-competent animals for the control PaCa 5061 and the ITGAV KD group, respectively). After a growth period of 62 days, the ITGAV KD alone almost completely abolished intraperitoneal carcinomatosis in wild-type mice. Only 7 of 15 mice (47%) showed any sign of tumor development (small tumors at the injection site with only one animal displaying macroscopically visible intraperitoneal carcinomatosis (with a minimal PCI of 1.) In the experimental group with control PaCa 5061 cells injected into selectin-deficient mice, 11 of 12 mice showed a tumor take (92%), with all of them displaying intraperitoneal carcinomatoses (mean PCI of 12.92; *P* <  0.001, Fig. 3a). After 62 d, only 2 of 12 selectin k.o. mice (17%) injected with ITGAV KD cells displayed any small tumors at the injection site and no carcinomatosis was observed. Due to this, the presumed synergistic effect of the selectin knockout could not be determined. Therefore, we extended the experimental time to 77 days and demonstrated a synergistic effect of ITGAV knockdown and selectin knockout (13 and 14 selectin-deficient *Pfp*^*−/−*^/*Rag2*^*−/−*^ for the control cells and the ITGAV KD cells, respectively, *P* = 0.017, Fig. 3c). Tumor take rates were as follows: selectin k.o. mice injected with control cells: 8 of 13 (62%) and k.o. mice injected with ITGAV KD cells: 6 of 14 (43%). Of the latter, half of the mice developed no peritoneal carcinomatosis, while in the remaining half, only minimal peritoneal carcinomatosis was observed, resulting in a mean PCI of 0.67. In contrast, injection of ITGAV KD cells into wild-type mice led to a mean PCI of 3.13 after 77 days, with at least small macroscopically visible tumor masses being present in all eight animals with tumor take.

As mentioned above, PaCa 5061 cells formed subcutaneous primary tumors in and around the injection channel after intraperitoneal injection: tumor weight in the group of wild-type mice with ITGAV KD cells was significantly reduced (mean of 116 mg, *P* = 0.003) and a synergistic effect could be observed for the selectin-deficient animals with ITGAV KD cells. Here, tumor weights of the injection channel tumors (mean of 22 mg, *P* = 0.017) were again significantly reduced compared with their wild-type counterparts (Fig. 3b).

Due to the different sizes of injection-site tumors of ITGAV KD and control cells in selectin-deficient and wild-type mice, we expected a functional effect of ITGAV on primary tumor development. To verify this observation, we performed an additional subcutaneous xenograft experiment with ITGAV KD and control cells in selectin wild-type mice. In other cancer entities, it could be shown that ITGAV activates latent TGF-β in the extracellular matrix (ECM) [19]. As SMAD4 is part of the transforming growth factor-β (TGF-β) signaling pathway and is inactivated in 50% of PDA patients [20] – resulting in dysfunctional canonical TGF-β signaling in the corresponding tumors – we additionally included the cell line BxPC3 with SMAD4 deletion as a model cell line for this patient subset. SMAD4 deletion was confirmed by absent SMAD4 protein levels in the case of BxPC3 (Fig. 3d). Again, sublines with and without stable knockdown of ITGAV, designated BxPC3 ITGAV KD and BxPC3 control, were generated. The expression of ITGAV was reduced by > 65% in the KD cell line compared to the expression levels of the control cells (Fig. 2).

After subcutaneous tumor cell injection (10 pfp^−−^/rag2^−−^ mice each s.c. injected with control and ITGAV KD PaCa 5061, respectively), 9 of 10 animals (90%) injected with control cells and 7 of 10 (70%) mice injected with ITGAV KD cells developed s.c. tumors until the experiment was terminated after 199 days. Mice inoculated with PaCa 5061 ITGAV KD cells showed a significantly prolonged overall survival until reaching the termination criteria (median survival 198.5 vs. 115 d for the control cells, *P* = 0.01, Fig. 3e). Similar results were found for BxPC3 (median survival 74 vs. 99 d, *P* <  0.001, Fig. 3h). All animals injected with BxPC3 cells (11 with control and 10 with ITGAV KD) developed s.c. tumors, except one animal from the ITGAV KD group, found dead 1 week after injection. It showed no sign of tumor development and was thus excluded from all further analyses. As tumor size and weight at the time of death did not differ significantly between the ITGAV and control groups for both cell lines, a significantly reduced growth of the ITGAV KD tumors can be assumed for PaCa 5061 and BxPC3 cells.

To analyze the influence of ITGAV knockdown on hematogenous metastasis, tumor cells in the animals’ lungs and blood were quantified for all mice by qRT-PCR. The PaCa 5061 ITGAV KD (*P* = 0.045, Fig. 3f) and BxPC3 ITGAV KD (*P* = 0.026, Fig. 3i) groups showed significantly less human DNA in the lungs, reflecting significantly fewer PDA cells than the control group. The numbers of circulating tumor cells in the animals’ peripheral blood decreased due to the ITGAV KD, albeit not reaching statistical significance (Fig. 3g and j).

### Effects of ITGAV knockdown in PaCa 5061 und BxPC3 cells in vitro

The possible integrin-beta subunits corresponding with ITGAV (ITGB1, ITGB3, ITGB5, ITGB6 and ITGB8) were determined in vitro using flow cytometry. Of these, only ITGB1 and ITGB6 were relevantly expressed in PaCa5061 and BxPC3 cells. Subunits ITGB1 and ITGB6 were found to be downregulated on PaCa 5061 ITGAV KD cells in vitro (Fig. 2b)*.* In contrast, only ITGB6 was downregulated on BxPC3 ITGAV KD cells, while the expression of ITGB1 remained almost unchanged (Fig. 2b). These changes were confirmed for the xenograft tumors by western blot (Fig. 2c) and immunohistochemistry (Fig. 6).

**Fig. 4:**
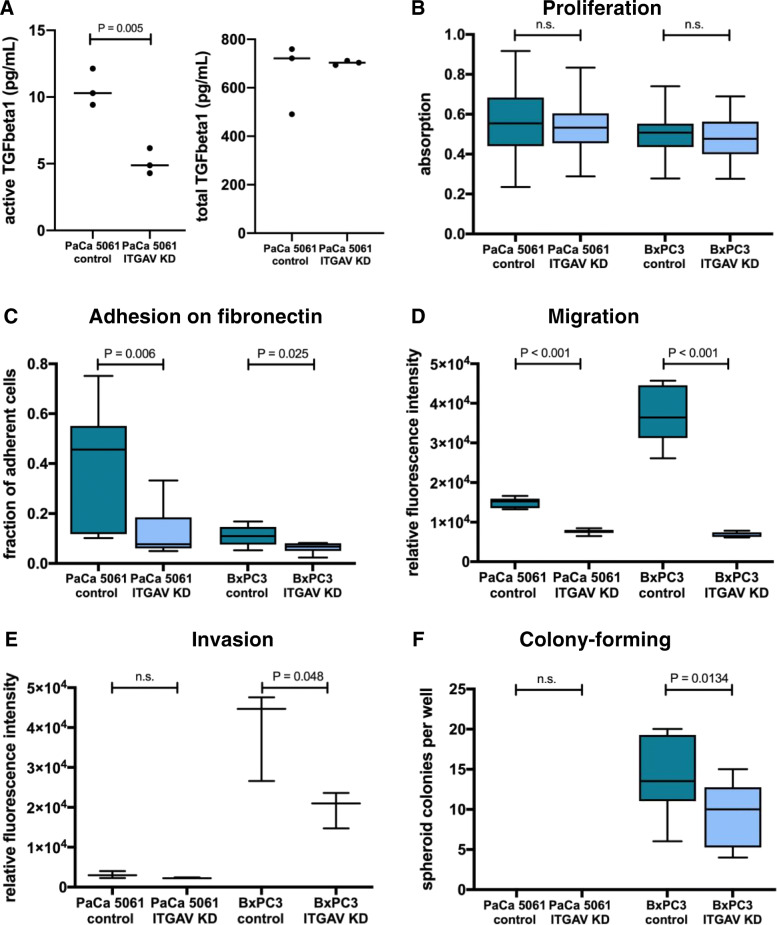
Concentration of active TGF-β1 and total TFG-β1 measured in the cell culture supernatant of PaCa 5061 ITGAV KD cells and control cells using ELISA (**a**). The proliferation assay showed no significant differences between the ITGAV knockdown groups and the corresponding control groups (**b**). The static adhesion on fibronectin was reduced by ITGAV knockdown (**c**). The migratory potential of tumor cells was decreased by the ITGAV KD (**d**), while the invasive potential was only impaired in the case of BxPC3 cells (**e**). In the colony-forming assay, BxPC3 ITGAV KD cells formed fewer spheroid colonies than the control cells. PaCa 5061 cells were not able to form colonies in Matrigel after 14 days (**f**)

To elucidate whether ITGAV activates TGF-β1 in vitro, an ELISA was used to determine the concentration of active and latent TGF-β1 in supernatants from cell culture medium. The concentration of active TGF-β1 decreased by 57% to 5.11 pg/mL (*P* = 0.005, Fig. 4a), whereas the concentration of total TGF-β1 did not differ significantly. Obviously, ITGAV activates latent TGF-β1 for PaCa 5061 cells in vitro*.*

To characterize the functional effects of ITGAV KD in vitro, proliferation, adhesion, migration, invasion and colony-forming assays were performed. No significant differences were detected in cell proliferation between knockdown and control cells in vitro as determined by XTT assays (Fig. 4b). In line with this finding, in vivo proliferation rates analyzed by determining the Ki-67 staining index of vital xenograft tumor tissue revealed no significant differences between the groups (Fig. 6).

The basal membrane / extracellular Matrix (ECM) lies open between the peritoneal cells [21], which might enable direct contact between ITGAV on the intraperitoneal tumor cells and its ligands such as fibronectin. Adhesion of ITGAV KD cells on fibronectin was analyzed under static conditions: PaCa 5061 cells with ITGAV KD showed a distinctly reduced number of adhering cells, with 11% compared with 39% for the controls (*P* = 0.006). Likewise, a reduction in the adhesive potential of BxPC3 cells could be demonstrated (*P* = 0.025, Fig. 4c).

The proportion of migrating tumor cells was reduced for both cell lines upon ITGAV KD (*P* <  0.001, Fig. 4d). Moreover, BxPC3 ITGAV knockdown cells showed a reduced invasive potential compared with the control cells (*P* = 0.048). PaCa 5061 cells were not able to pass the Matrigel layer at all (Fig. 4e). In line with the invasion assay finding, BxPC3 ITGAV KD cells formed fewer spheroid colonies per well than the control cells (*P* = 0.0134, Fig. 4f). Representative images of colonies formed by BxPC3 control and BxPC3 ITGAV KD cells are shown in Supplementary Fig. 1. PaCa 5061 cells were not able to form colonies in Matrigel after 14 days.

### Gene expression and immunohistochemical analysis of the primary tumors

**Fig. 5:**
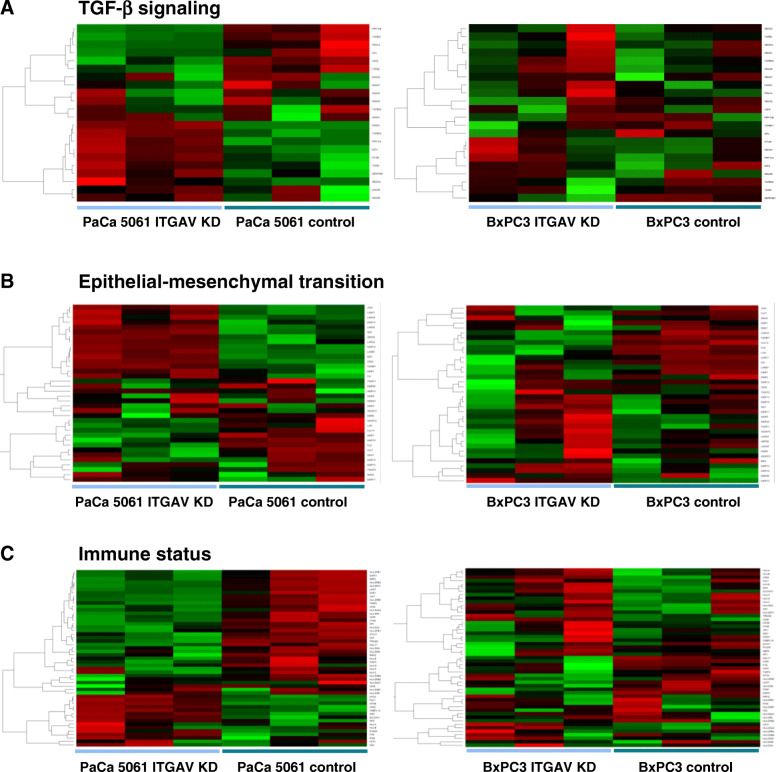
Heat map and clustering of gene expression data for PaCa 5061 and BxPC3 xenograft tumors: TGF-β signaling (**a**), epithelial-mesenchymal transition (**b**) and immune status (**c**). A significantly higher expression is shown in red and a lower expression in green. In contrast to the gene expression changes in BxPC3 xenograft tumors (no canonical signaling due to the deletion of SMAD4), changes in PaCa 5061 (SMAD4 intact) xenograft tumors appeared organized

**Fig. 6:**
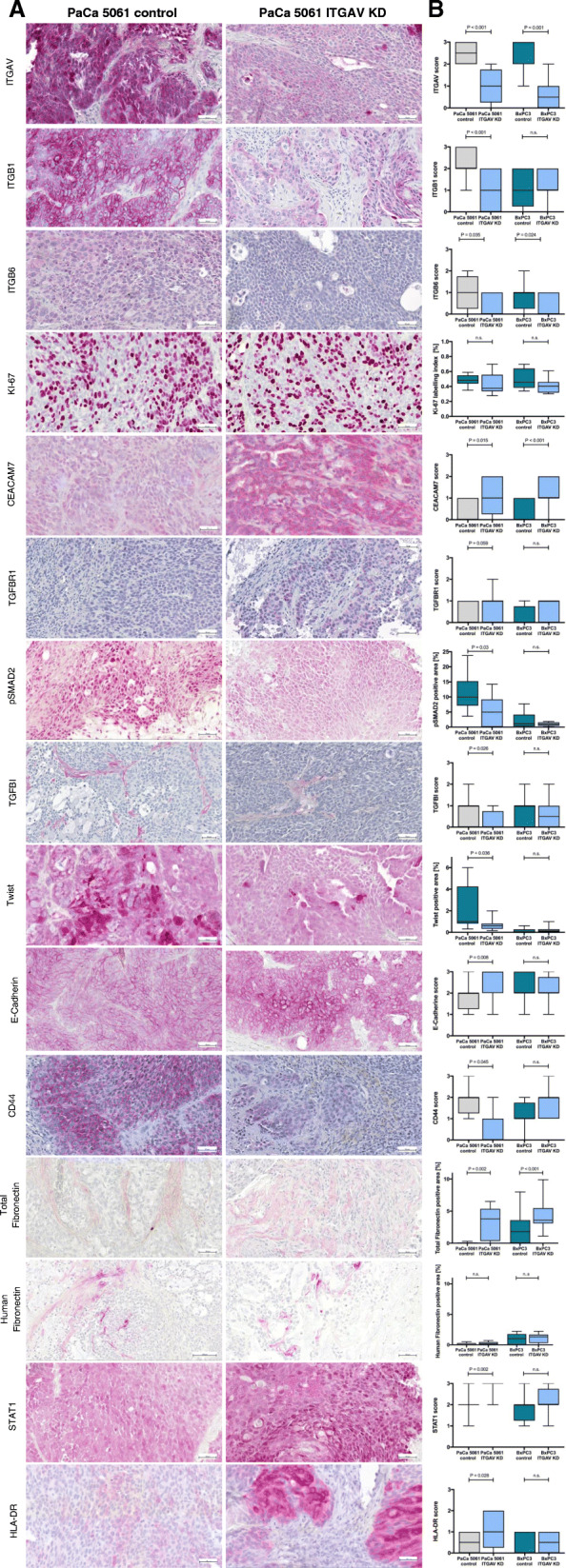
Representative images of immunohistochemical staining of ITGAV, ITGB1, ITGB6, Ki-67, CEACAM7, TFBR1, pSMAD2, pSMAD2, TGFBI, Twist, E-cadherin, CD44, total and human fibronectin, STAT1 and HLA-DR (**a**) and quantification (**b**). The shown images are representative of three tumors harvested

To gain insight into how the ITGAV knockdown might cause the reduction in tumor growth and metastasis/carcinomatosis formation observed in vivo, we performed whole human genome expression analyses. Genes of interest were defined and categorized into groups according to their functional context (Table 1, Fig. 5). Differences in selected proteins were validated by immunohistochemistry (Fig. 6).

**Table 1:** Selected gene expression changes in PaCa 5061 xenograft tumors upon ITGAV KD. FDR adjusted *P*-values < 0.10 are highlighted (bold font)

Gene Symbol	Fold Change	P	FDR adj. P
**TGF-β signaling**			
CDK8	1.8	0.007	0.108
E2F4	**−2.3**	**< 0.001**	**0.007**
LTBP1	**− 2.61**	**0.0003**	**0.0201**
NEDD4L	−1.3	0.096	0.462
PARD3	**− 2.43**	**< 0.001**	**0.001**
PPM1A	**2.42**	**0.002**	**0.051**
PPP1CA	**−1.55**	**< 0.001**	**0.013**
PPP1CB	**2.59**	**< 0.001**	**0.003**
RAP1B	1.5	0.0064	0.1065
SERPINE1	**−2.83**	**0.004**	**0.077**
SKIL	**2.92**	**0.002**	**0.051**
SMAD1	−1.18	0.310	0.733
SMAD2	1.09	0.809	0.958
SMAD3	1.79	0.273	0.704
SMAD4	−1.08	0.917	0.982
SMAD6	−1.13	0.289	0.717
SMAD7	1.06	0.505	0.851
SMAD9	−1.01	0.595	0.887
STUB1	**−1.84**	**< 0.001**	**0.019**
TGFB1	−1.59	0.014	0.170
TGFB2	**3.61**	**0.003**	**0.071**
TGFBI	**−0.11**	**0.065**	**0.384**
TGFBR1	**1.68**	**< 0.001**	**0.015**
TGFBR2	1.22	0.971	0.994
TGFBR3	**−2.75**	**0.005**	**0.090**
**Epithelial-mesenchymal transition**			
ACTA2	−1.04	0.6051	0.8912
ADAM10	**2.26**	**0.003**	**0.063**
ADAM12	1.01	0.897	0.979
BSG	**−2.65**	**< 0.001**	**0.002**
CDH1	−1.21	0.3769	0.7813
CDH2	**−5.04**	**< 0.001**	**< 0.001**
FGFBP1	−1.86	0.007	0.107
FLG	**265.8**	**< 0.001**	**0.002**
FN1	**−2.57**	**0.002**	**0.048**
HSPG2	**−1.78**	**0.001**	**0.039**
JAG1	**−3.05**	**< 0.001**	**0.005**
KLK13	**2.06**	**0.001**	**0.043**
KLK7	3.18	0.008	0.125
LAMA2	**−1.61**	**0.001**	**0.028**
LAMA3	**−6.57**	**< 0.001**	**0.003**
LAMA5	−1.74	0.072	0.404
LAMB1	**−4.02**	**< 0.001**	**< 0.001**
LAMC1	**−1.99**	**0.001**	**0.039**
LUM	1.14	0.249	0.682
MMP1	−2.59	0.160	0.577
MMP10	**−7.92**	**< 0.001**	**0.001**
MMP12	1.29	0.918	0.983
MMP13	−1.42	0.200	0.629
MMP14	−1.73	0.008	0.124
MMP15	1.01	0.960	0.992
MMP16	1.09	0.175	0.598
MMP17	−1	0.851	0.968
MMP2	1.06	0.159	0.576
MMP20	1.01	0.562	0.875
MMP24	1.03	0.990	0.998
MMP3	1.07	0.635	0.903
MMP7	**2.95**	**< 0.001**	**0.010**
MMP9	−1	0.945	0.990
MUC4	5.58	0.001	0.0366
NID1	**−2.3**	**< 0.001**	**0.009**
OCLN	**17.35**	**< 0.001**	**0.010**
POSTN	1.03	0.1091	0.4901
SNAI1	1.19	0.107	0.486
SNAI2	−1.11	0.459	0.828
SPP1	1.08	0.9279	0.9849
TWIST1	1.08	0.313	0.734
TWIST2	1.09	0.203	0.633
VIM	1.2	0.1338	0.5344
**Immune status**			
CD2	−1.08	0.312	0.734
CD4	**2.46**	**< 0.001**	**0.002**
CD46	**2.88**	**0.001**	**0.029**
CD48	1.05	0.464	0.830
CD55	1.75	0.016	0.177
CD74	−1.05	0.891	0.978
CNN2	**−1.62**	**0.001**	**0.046**
CTSS	**3.59**	**0.001**	**0.033**
DAPK1	**3.58**	**0.001**	**0.027**
FYB	−5.95	0.036	0.279
GBP2	**7.67**	**< 0.001**	**0.012**
HLA-A	−1.09	0.306	0.730
HLA-B	−1.13	0.439	0.818
HLA-C	1.03	0.745	0.941
HLA-DMA	1.58	0.007	0.110
HLA-DMB	1.41	0.009	0.129
HLA-DOA	1.26	0.009	0.129
HLA-DOB	1.09	0.437	0.817
HLA-DPA1	**3.32**	**< 0.001**	**0.005**
HLA-DPB1	1.4	0.019	0.197
HLA-DPB2	1.21	0.031	0.257
HLA-DQA1	−1.14	0.294	0.721
HLA-DQA2	1.77	0.008	0.123
HLA-DQB1	−1.14	0.492	0.845
HLA-DQB2	**1.41**	**0.003**	**0.065**
HLA-DRA	**14.81**	**< 0.001**	**0.004**
HLA-DRB1	**19.95**	**0.001**	**0.025**
HLA-DRB4	**12.38**	**< 0.001**	**0.005**
HLA-DRB5	**3.45**	**< 0.001**	**0.002**
HLA-E	1.17	0.199	0.628
HLA-G	1.64	0.048	0.328
HLA-H	1.13	0.298	0.724
ICAM1	1.71	0.065	0.385
IFNG	−1.07	0.179	0.603
IRF1	**1.37**	**0.005**	**0.094**
IRF2	− 1.56	0.014	0.170
JAK1	**1.82**	**< 0.001**	**0.015**
KIF3B	−1.73	0.004	0.076
LMO7	**5.19**	**< 0.001**	**0.005**
MALT1	**1.72**	**0.003**	**0.069**
MID1	**−3.3**	**< 0.001**	**0.007**
MT2A	**−2.36**	**0.002**	**0.049**
OAS1	1.78	0.014	0.169
OSBPL1A	**−2.52**	**< 0.001**	**0.010**
PAK1	**−2.66**	**< 0.001**	**0.004**
PTAFR	−2.09	0.006	0.102
RIPK2	2.56	0.020	0.205
SLCO4C1	**−2.93**	**0.001**	**0.040**
STAT1	**2.58**	**< 0.001**	**0.019**
TIMP2	**4.83**	**< 0.001**	**0.008**
TRIM22	**2.28**	**< 0.001**	**0.004**

Starting with the SMAD4-intact PaCa 5061 cells, changes regarding the TGF-β signaling are compiled first: The non-signaling reservoir TGF-β receptor 3 (TGFBR3) was found to be downregulated in the ITGAV KD tumors. In contrast, the expression of TGF-β2 (activation not dependent on ITGAV) and TGF-βR1 (receptor for TGF-β1, 2 and 3) were upregulated. Expression of the fibronectin-anchored, latent-transforming growth factor beta-binding protein 1 (LTBP1), which is essential for activating TGF-β3 with integrin αvβ6 [22], was downregulated in PaCa 5061 ITGAV KD tumors. Regarding the regulation of the SMAD2/3:SMAD4 complex, expression levels of several involved genes were significantly regulated, including E2F4, PPM1A, SERPINE1, SKIL and STUB1. To confirm the changed activity of TGF-β activation, staining for phospho-SMAD2 was performed (Fig. 6): The proportion of phospho-SMAD2-positive tumor cell nuclei was significantly higher in the PaCa 5061 control group compared with the ITGAV KD group (*P* = 0.03, Fig. 6b). Besides, transforming growth factor beta-induced (TGFBI), located in the ECM and associated with poor survival [23], is downregulated as a result of the ITGAV knockdown, which was again immunohistochemically confirmed (Fig. 6b).

Secondly, changes regarding epithelial-mesenchymal transition (EMT) were as follows: Weaker intranuclear staining signals for Twist, a master regulator of EMT, which is controlled by TGF-β1 [24], was observed in PaCa 5061 ITGAV KD tumors (Fig. 6b). In line with the KD cells’ more epithelial phenotype, Occludin was found to be 17.35-fold upregulated in the PaCa 5061 ITGAV KD tumors; accordingly, E-cadherin staining signals were enhanced in the ITGAV KD tumors (Fig. 6b). CD44, which correlated with EMT and poor survival in a clinical study of PDA [25], was downregulated 8.94-fold upon knockdown of ITGAV. The secretion of matrix proteins and proteases was also found to be modulated to favor tumor progression in PDA [19]. Exemplarily, MMP-10 was decreased upon knockdown of ITGAV accompanied by increased stromal (murine) fibronectin: Staining for both human and murine fibronectin using a cross-reactive antibody revealed a moderate signal in the ITGAV knockdown groups, whereas the control groups were predominantly only weakly stained for fibronectin (Fig. 6). In contrast, specific staining for human fibronectin revealed no differences between the two groups, indicating that the stromal fibronectin was deposited by murine stromal cells and not by human cancer cells.

Thirdly, changes regarding the immune status were as follows: MHC class II molecules, part of the human leukocyte antigen (HLA) system, were upregulated in the ITGAV KD xenograft tumors (i.e. HLA-DRA, HLA-DRB1, HLA-DRB4, HLA-DRB5, HLA-DPA1 and HLA-DQB2). Exemplarily, we verified the upregulation of HLA-DR by immunohistochemistry (Fig. 6). Additionally, the upregulation of HLA-DR on PaCa 5061 ITGAV KD cells was also confirmed in vitro using flow cytometry (Supplementary Fig. 2). Increased JAK-STAT signaling leads to increased expression of MHC class II genes [26] and, indeed, both JAK1 and STAT1 were found to be increased in the ITGAV KD xenografts. Using immunohistochemistry, an increased intranuclear STAT1 signal could also be confirmed in the corresponding ITGAV KD xenograft tumors at the protein level (Fig. 6).

When gene expression profiles of the SMAD4-deficient BxPC3 xenografts with knockdown of ITGAV were analyzed, no genes associated with TGF-β signaling, EMT or immune status were found to be significantly regulated. We attribute this gene expression pattern to the malfunction of the canonical TGF-β signaling pathway caused by the confirmed deletion of SMAD4 (Fig. 3d).

Finally, ITGAV targets concordantly altered in both PaCa5061 and BxPC3 xenograft tumors could be assigned to the group of cell surface interactions (s. Supplementary Table 1). E.g., the adhesion molecule CEACAM7 was significantly upregulated in both models, as demonstrated by immunohistochemistry (Fig. 6).

### Clinical proof of concept using a tissue microarray

**Fig. 7:**
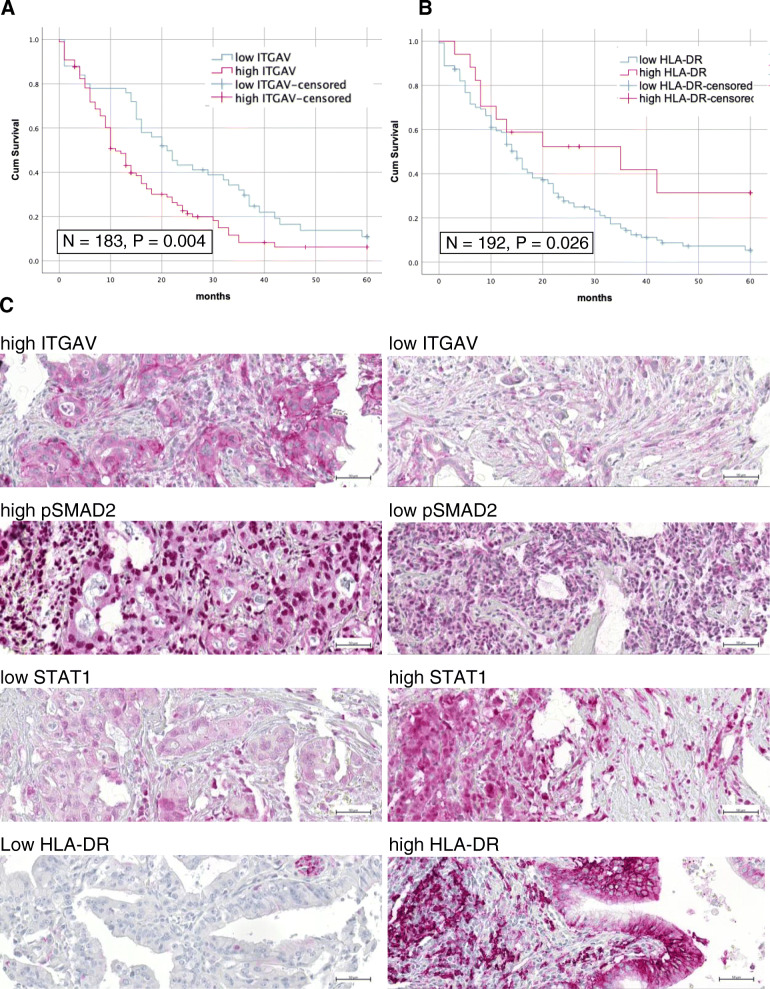
Patients with high expression of ITGAV (*N* = 129) had a significantly poorer survival with a mean survival of 11 months than patients with low expression of ITGAV (*N* = 54) with a mean survival of 21 months (**a**). Patients with high expression of HLA-DR (*N* = 22) showed a significantly better survival with a mean survival of 35 months than patients with low expression of HLA-DR (*N* = 170) with a mean survival of 15 months (**b**). Representative tissue samples are given (**c**). Scale bar: 50 μm

A high level of ITGAV expression in the PDA cells was found in 129 tissue samples (71%). These patients showed a significantly poorer outcome with a median survival of 11 (95% CI 8.6–13.3) months instead of 21 (95% CI 15.5–26.5) months in the group of patients with low ITGAV expression (*P* = 0.004, Fig. 7a). For 125 tissue samples (69%), a high percentage of pSMAD2-positive tumor cell nuclei was identified and ITGAV and phospho-SMAD2 staining intensities were positively correlated (Supplementary Table 2, *P* = 0.014). However, there was no significant correlation for phospho-SMAD2 with survival (*P* = 0.127). Ninety three tissue samples (58%) displayed a high expression of STAT1. No significant association existed between STAT1 and survival (*P* = 0.310). High expression of HLA-DR was found in 22 tumor samples. Of these 22 tumors, 17 were also STAT1 positive. Hence, a positive correlation between STAT1 and HLA-DR expression was observed (Supplementary Table 2, *P* = 0.019). This observation supports the connection between JAK-STAT signaling and the expression of HLA-DR. In our cohort, a better survival could be demonstrated for patients with high expression of HLA-DR by tumor cells with a median survival of 35 (95% CI 0.6–69.4) months compared with 15 (95% CI 12.9–17.1) months for patients with low HLA-DR expression (Fig. 7b). None of the staining results showed any correlation with gender, grading or tumor stage (Supplementary Table 2).

## Discussion

In the present study, we could show that ITGAV expression was upregulated in intraperitoneal pancreatic ductal adenocarcinoma xenograft tumor cells that grew under selectin-deficient conditions, suggesting a compensatory increase in integrin expression. We could demonstrate that the knockdown of ITGAV led to a massive reduction in intraperitoneal carcinomatosis, primary tumor growth and pulmonary metastasis. Even more importantly, the effects of the absence of host selectins and ITGAV were synergistic, as clearly demonstrated in our intraperitoneal tumor model. This is the first model to describe such a synergism experimentally. These findings validate the hypothesis that cancer cell adhesion to mesothelial cells follows similar mechanisms as the leukocyte adhesion cascade, which is initiated by selectins followed by integrins [27]. Integrin-mediated cell adhesion also seems to be essential in compensating for the absence of selectin expression [28]. The observed increased expression in other cell adhesion molecules in the ITGAV KD tumors (e.g. the adhesion molecule CEACAM-7) might partly compensate for the reduction of ITGAV. Our findings indicate that the adhesion to the peritoneal mesothelium or its underlying basal lamina is the rate-limiting step of peritoneal carcinomatosis formation in PDA. Fortunately, this step is particularly amenable to therapeutic invention as the molecules involved are located on the cell surface. Therefore, blockage of both selectins and integrins is an attractive option to inhibit peritoneal metastasis in PDA.

A body of literature suggests that ITGAV activates latent TGF-β in the ECM [19, 29]. We could demonstrate this for the SMAD4-intact PaCa 5061 cells, for which the concentration of active TGF-β1 is reduced due to the knockdown of ITGAV in vitro. Furthermore, the knockdown of ITGAV reduced the activity of phospho-SMAD2 in the xenograft tumors of the SMAD4-intact PaCa 5061 cells in vivo. Our gene expression analyses confirmed altered TGF-β-signaling due to the knockdown of ITGAV in PaCa 5061 tumors, as described above. Moreover, there is an association between ITGAV and pSMAD2-positive tumors in the analyzed patient samples: high expression of ITGAV is connected with poorer survival. This observation corroborates data in the GEPIA2 webserver (Supplementary Fig. 3, [30]) and Human Protein Atlas [31], which show a correlation between ITGAV mRNA expression level and poor patient survival. Moreover, a recent study demonstrated that elevated serum soluble TGF-β predicted poor survival in PDA [32].

At the beginning of the tumorigenic process, TGF-β1 functions as a tumor suppressor due to its ability to suppress cell division in epithelial cells [33]. Hezel et al. used a genetically engineered mouse model with Kras^G12D^-initiated, SMAD4-deficient murine PDAs reflecting an early disease state and identified increased tumor cell proliferation through the blockade of integrin αvβ6 [34]. The cell lines used in our study originate from locally advanced human tumors, as commonly present at the time of diagnosis. Our proliferation assay in vitro and the determination of the Ki-67 Labeling Index in the xenograft tumors show no changes in cell proliferation (Fig. 5b), suggesting that the examined cell lines are already resistant to the anti-proliferative effects of TGF-β.

Bates et al. reported that a high level of integrin αvβ6 is accompanied by a poorer overall survival for colon carcinoma. They assumed the reason for this to be the integrin αvβ6-dependent activation of TGF-β1 and the resulting stimulation of EMT [35]. In PDA, high integrin αvβ6 mRNA levels were associated with shortened patient survival and antibody therapy directed against this dimer suppressed the pro-tumorogenic microenvironment (e.g. by suppression of TGF-β signaling) in mouse models [7]. Besides the critical alterations induced by ITGAV in TGF-β signaling for SMAD4 intact PDA (represented by PaCa 5061 in this study), the xenograft model with the SMAD4 dysfunctional BxPC3 cells demonstrates that there are also TGF-β independent effects of ITGAV in PDA. The TMA data further corroborate this observation: Although Tumors were not stratified for functional SMAD4, ITGAV expression was still prognostic in the overall cohort. Together with the fact that half of all PDA display deletions / inactivating mutations of SMAD4 [20], this indicates that ITGAV is prognostic for SMAD4 functional as well as dysfunctional tumors. In prostate cancer, for example, AKT activation has been described as an additional mechanism for ITGAV involvement in a recent study [4]. Our study shows that the proportion of Twist-positive cells decreases through the knockdown of ITGAV in the SMAD4-intact PaCa 5061 cells. Twist controls the expression of epithelial gene signatures such as Occludin and E-cadherin [36] and indeed, expression of these epithelial genes increased upon ITGAV KD. In pharyngeal carcinoma, Van Aarsen et al. demonstrated that blocking with an integrin αvβ6 antibody reduced TGF-β-induced SMAD2 phosphorylation, which resulted in diminished tumor growth and reduced invasive potential while an impact of the treatment on cell proliferation was not observed [37]. These effects were confirmed for PDA in our study.

In vivo*,* TFG-β1 is a potent activator of the transdifferentiation of fibroblasts into myofibroblasts, which are referred to as cancer-associated fibroblasts (CAFs) [38]. The increase in the ECM contraction caused by CAFs increases the probability of TGF-β activation [38]. Interestingly, it has been shown that the heterogeneity of CAFs in PDA is mediated by JAK/STAT signaling antagonized by TGF-β [39]. CAFs themselves interact with integrins and secrete a large number of proteins into the ECM matrix, proteases and cytokines, which can further increase cancer progression [40]. For example, CAFs deposit fibronectin in the ECM, thereby creating pro-migratory tracks [41]. It is assumed that CAFs themselves express ITGAV and hence can also activate latent TGF-β [42]. Consistently, we found an upregulation of murine fibronectin in the xenograft tumors with ITGAV KD (see Fig. 5a and e).

Highly interesting is the immunomodulatory effect of the ITGAV knockdown found in our study, especially since results for immunotherapy of PDA [43] must be considered disappointing so far. To our knowledge, the observed changes in MHC-II expression by PDA have not been described before. However, to fully investigate the implications of these alterations, a suitable new in vivo model with an intact adaptive immune system would be needed, e.g. a syngenic model. As MHC-II is required for CD4+ T-cell activation, which also plays essential roles in antitumor immunity [44], further investigations in this field could prove highly rewarding.

## Conclusions

The ITGAV knockdown of PDA massively suppressed the intraperitoneal carcinomatosis of these cells. Moreover, the effects of the absence of selectins and reduced expression of ITGAV on intraperitoneal carcinomatosis are synergistic, confirming the hypothesis of a multistep and partly redundant leukocyte adhesion cascade as the rate-limiting step within the metastatic cascade. Mechanistically, ITGAV activates TGF-β and drives epithelial-mesenchymal transition PDA cells. Specific inhibition of ITGAV may have the potential to impede intraperitoneal carcinomatosis, tumor growth and distant metastasis.

## Supplementary Information

**Additional file 1: Supplementary Figure 1.** Representative images of colonies formed by BxPC3 control (A) and BxPC3 ITGAV KD (B) cells after 14 d. BxPC3 ITGAV KD cells formed fewer spheroid colonies per well than the control cells (*P* = 0.0134, Fig. 4F).

**Additional file 2: Supplementary Figure 2.** Changes HLA-DR using flow cytometry: HLA-DR is upregulated on PaCa 5061 ITGAV KD cells. The signal of HLA-DR on BxPC3 cells was very low.

**Additional file 3: Supplementary Figure 3.** For an in silico analysis of the association between gene expression and patient survival in the GEPIA2 webserver, we used the heterodimer ITGAV and ITGB6 as a signature. High expression of ITGAV and ITGB6 is associated with poor survival (*P* <  0.001).

**Additional file 4: Supplementary Table 1.** Changes in expression of genes involved in cell surface interaction of PaCa 5061 and BxPC3 xenograft tumors. **Supplementary Table 2.** Clinico-pathological data of patients with pancreatic adenocarcinoma correlated with ITGAV, pSMAD2, STAT1 and HLA-DR.

## Data Availability

The datasets used and/or analyzed during the current study are available from the corresponding author on reasonable request.

## Abbreviations

PDA: Pancreatic ductal adenocarcinoma
shRNA: Short hairpin RNA
ELISA: Enzyme-linked immunosorbent assay
PCI: Peritoneal carcinomatosis index
qRT-PCR: Quantitative real-time polymerase chain reaction
TMA: Tissue microarray
KD: Knockdown
ECM: Extracellular matrix
EMT: Epithelial-mesenchymal transition
HLA: Human leucocyte antigen
CAFs: Cancer-associated fibroblasts
