# Supplementary figures and images for: Integrin alpha-V is an important driver in pancreatic adenocarcinoma progression
Source: J Exp Clin Cancer Res. 2021 Jun 26;40:214. doi: 10.1186/s13046-021-01946-2 (PMC8235815; doi:10.1186/s13046-021-01946-2)

**Supplementary Figure 1**

**A BxPC3 control**

**
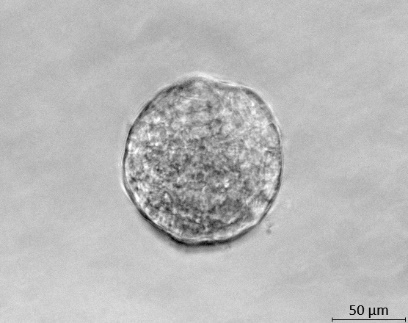

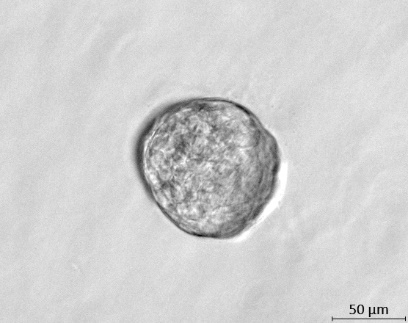

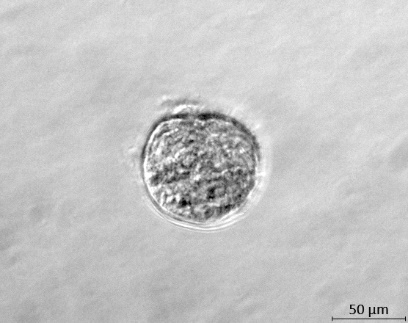
**

**B BxPC3 ITGAV KD**

**
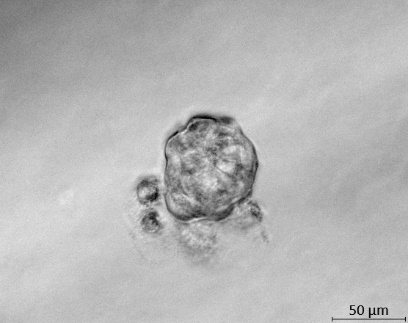

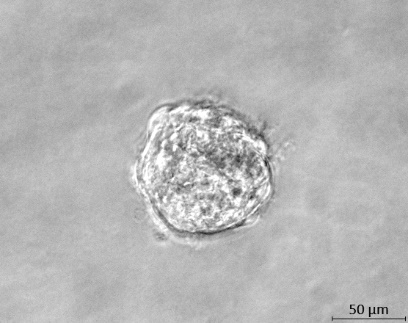

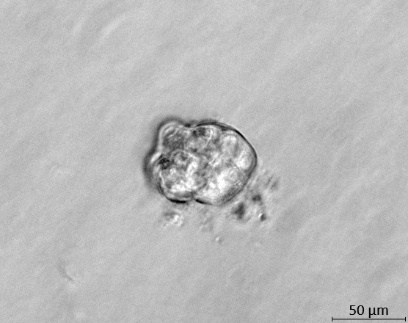
**

Supplement: Supplementary file 1 — Additional file 1: Supplementary Figure 1. Representative images of colonies formed by BxPC3 control (A) and BxPC3 ITGAV KD (B) cells after 14 d. BxPC3 ITGAV KD cells formed fewer spheroid colonies per well than the control cells (P = 0.0134, Fig. 4F). [file 13046_2021_1946_MOESM1_ESM.docx]

**Supplementary Figure 2**


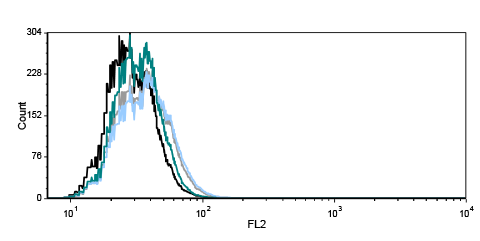

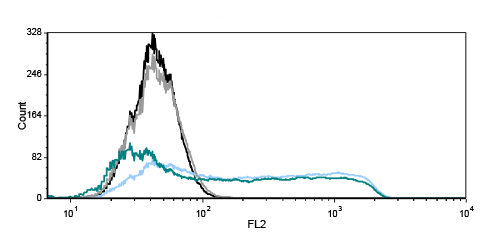
**A PaCa 5061 B BxPC3**

**Signal**

**ITGAV KD: 428.44**

**Control: 329.20**

**↑53%**

**HLA-DR**

**Signal**

**ITGAV KD: 39.58**

**Control: 34.58**

**↑78%**

Supplement: Supplementary file 2 — Additional file 2: Supplementary Figure 2. Changes HLA-DR using flow cytometry: HLA-DR is upregulated on PaCa 5061 ITGAV KD cells. The signal of HLA-DR on BxPC3 cells was very low. [file 13046_2021_1946_MOESM2_ESM.docx]

**Supplementary Figure 3**


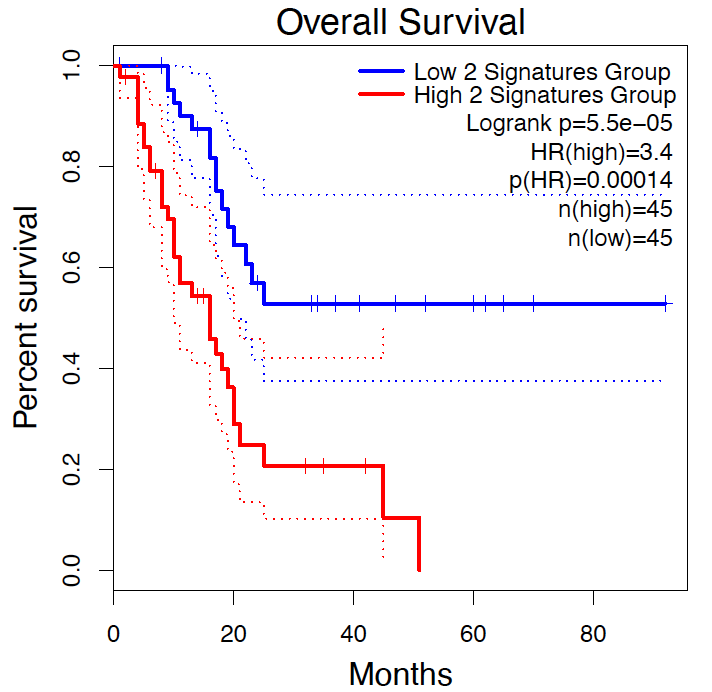

Supplement: Supplementary file 3 — Additional file 3: Supplementary Figure 3. For an in silico analysis of the association between gene expression and patient survival in the GEPIA2 webserver, we used the heterodimer ITGAV and ITGB6 as a signature. High expression of ITGAV and ITGB6 is associated with poor survival (P < 0.001). [file 13046_2021_1946_MOESM3_ESM.docx]
