# Supplementary material for: Integrin alpha-V is an important driver in pancreatic adenocarcinoma progression
Source: J Exp Clin Cancer Res. 2021 Jun 26;40:214. doi: 10.1186/s13046-021-01946-2 (PMC8235815; doi:10.1186/s13046-021-01946-2)
Supplement: Supplementary file 4 — Additional file 4: Supplementary Table 1. Changes in expression of genes involved in cell surface interaction of PaCa 5061 and BxPC3 xenograft tumors. Supplementary Table 2. Clinico-pathological data of patients with pancreatic adenocarcinoma correlated with ITGAV, pSMAD2, STAT1 and HLA-DR. [file 13046_2021_1946_MOESM4_ESM.docx]

**SUPPLEMENTARY DATA**

**Supplementary Table 1:** Changes in expression of genes involved in cell surface interaction of PaCa 5061 and BxPC3 xenograft tumors.

|  | **PaCa 5061** |  |  | **BxPC3** |  |  |
| --- | --- | --- | --- | --- | --- | --- |
| Gene Symbol | Fold Change | P | FDR adj. P | Fold Change | P | FDR adj. P |
| CD44 | **-8.94** | **< 0.001** | **0.007** | 1.21 | 0.492 | 0.987 |
| CEACAM1 | 2.94 | 0.027 | 0.242 | 2.58 | **0.017** | 0.886 |
| CEACAM3 | 1.03 | 0.387 | 0.787 | 1.13 | 0.249 | 0.958 |
| CEACAM5 | 7.07 | **0.024** | 0.224 | 2.79 | 0.184 | 0.944 |
| CEACAM7 | **9.7** | **0.003** | **0.062** | 7.56 | **0.002** | 0.582 |
| CEACAM8 | 1.21 | 0.435 | 0.816 | 1.56 | 0.065 | 0.907 |
| DSG3 | -2.17 | 0.102 | 0.475 | 4.5 | 0.022 | 0.887 |
| ITGA10 | 1.03 | 0.761 | 0.945 | 1 | 0.976 | 0.999 |
| ITGA11 | 1.04 | 0.480 | 0.839 | 1.01 | 0.936 | 0.999 |
| ITGA2 | -1.51 | 0.883 | 0.976 | 1.45 | 0.107 | 0.910 |
| ITGA2B | 1.01 | 0.787 | 0.952 | 1.05 | 0.492 | 0.987 |
| ITGA3 | **-1.91** | **< 0.001** | **0.012** | 1.39 | 0.408 | 0.979 |
| ITGA4 | 1.08 | 0.469 | 0.833 | 1.04 | 0.831 | 0.999 |
| ITGA5 | 1.23 | 0.681 | 0.920 | 2.69 | **0.005** | 0.778 |
| ITGA6 | -2.39 | **0.034** | 0.271 | 1.91 | 0.012 | 0.864 |
| ITGA7 | 1.22 | 0.182 | 0.608 | 1.05 | 0.972 | 0.999 |
| ITGA8 | 1.03 | 0.811 | 0.958 | 1.03 | 0.571 | 0.992 |
| ITGA9 | 1.07 | 0.543 | 0.867 | 1.02 | 0.382 | 0.974 |
| ITGAM | -1.23 | 0.028 | 0.248 | 1.03 | 0.848 | 0.999 |
| ITGB1 | -1.29 | 0.265 | 0.697 | 1.55 | 0.114 | 0.913 |
| ITGB2 | 1.04 | 0.706 | 0.929 | -1.06 | 0.563 | 0.991 |
| ITGB3 | **1.46** | **< 0.001** | **0.010** | -1.02 | 0.934 | 0.999 |
| ITGB5 | 1.19 | 0.437 | 0.817 | 1.25 | **0.084** | 0.910 |
| ITGB6 | 1.7 | **0.010** | 0.140 | 1.54 | **0.024** | 0.887 |
| ITGB7 | 1.12 | **0.034** | 0.271 | 1.08 | **0.078** | 0.910 |
| ITGB8 | 1.33 | **0.035** | 0.273 | 2.31 | **0.012** | 0.864 |
| OCLN | **17.35** | **< 0.001** | **0.010** | -1.01 | 0.596 | 0.992 |
| PDPK1 | -1.66 | **0.008** | 0.119 | -1.23 | 0.714 | 0.996 |
| RAP1A | **2.34** | **< 0.001** | **0.011** | -1.23 | 0,5672 | 0,9912 |
| ROCK1P1 | **-3.31** | **<0.001** | **0.015** | 1,26 | 0,8422 | 0,9992 |

**Supplementary Table 2**: Clinico-pathological data of patients with pancreatic adenocarcinoma correlated with ITGAV, pSMAD2, STAT1 and HLA-DR.

|  | **low**  **ITGAV** | **High**  **ITGAV** | **P** | **Low pSMA2** | **High pSMA2** | **P** | **Low STAT1** | **High STAT1** | **P** | **Low HLA-DR** | **High**  **HLA-DR** | **P** |
| --- | --- | --- | --- | --- | --- | --- | --- | --- | --- | --- | --- | --- |
| **Gender** |  |  | 0.297 |  |  | 0.426 |  |  | 0.174 |  |  | 0.137 |
| **Male** | 33 | 68 |  | 33 | 67 |  | 39 | 44 |  | 98 | 9 |  |
| **Female** | 21 | 61 |  | 22 | 58 |  | 28 | 49 |  | 72 | 13 |  |
| **pT** |  |  | 0.866 |  |  | 0.345 |  |  | 0.950 |  |  | 0.551 |
| **pT1** | 2 | 5 |  | 2 | 4 |  | 2 | 3 |  | 5 | 2 |  |
| **pT2** | 13 | 38 |  | 12 | 37 |  | 20 | 24 |  | 48 | 6 |  |
| **pT3** | 37 | 80 |  | 40 | 76 |  | 42 | 61 |  | 109 | 13 |  |
| **pT4** | 2 | 6 |  | 1 | 8 |  | 3 | 5 |  | 8 | 1 |  |
| **pN** |  |  | 0.078 |  |  | 0.874 |  |  | 0.366 |  |  | 0.865 |
| **pN0** | 25 | 42 |  | 20 | 47 |  | 27 | 31 |  | 65 | 8 |  |
| **pN1** | 29 | 87 |  | 35 | 78 |  | 40 | 62 |  | 105 | 14 |  |
| **pM** |  |  | 0.215 |  |  | 0.853 |  |  | 0.225 |  |  | 0.090 |
| **M0** | 53 | 121 |  | 52 | 119 |  | 62 | 90 |  | 162 | 19 |  |
| **M1** | 1 | 8 |  | 3 | 6 |  | 5 | 3 |  | 8 | 3 |  |
| **Grading** |  |  | 0.922 |  |  | 0.642 |  |  | 0.701 |  |  | 0.522 |
| **G1** | 2 | 5 |  | 2 | 4 |  | 3 | 2 |  | 9 | 0 |  |
| **G2** | 26 | 57 |  | 22 | 60 |  | 29 | 42 |  | 75 | 11 |  |
| **G3** | 26 | 56 |  | 30 | 60 |  | 34 | 48 |  | 84 | 11 |  |
| **AJCC classification** |  |  | 0.384 |  |  | 0.926 |  |  | 0.289 |  |  | 0.563 |
| **IA** | 1 | 4 |  | 1 | 3 |  | 0 | 3 |  | 4 | 1 |  |
| **IB** | 7 | 14 |  | 7 | 15 |  | 10 | 9 |  | 20 | 3 |  |
| **IIA** | 16 | 22 |  | 12 | 26 |  | 16 | 17 |  | 38 | 3 |  |
| **IIB** | 27 | 76 |  | 31 | 68 |  | 34 | 56 |  | 93 | 11 |  |
| **III** | 2 | 5 |  | 1 | 7 |  | 2 | 5 |  | 7 | 1 |  |
| **IV** | 1 | 8 |  | 3 | 6 |  | 5 | 3 |  | 8 | 3 |  |
| **ITGAV** |  |  |  |  |  | **0.014** |  |  | 0.607 |  |  | 0.578 |
| **Low** | - | - |  | 23 | 30 |  | 19 | 24 |  | 46 | 5 |  |
| **High** | - | - |  | 30 | 91 |  | 44 | 67 |  | 109 | 16 |  |
| **STAT1** |  |  | 0.607 |  |  | 0.680 |  |  |  |  |  | **0.019** |
| **Low** | 19 | 44 |  | 21 | 41 |  | - | - |  | 61 | 4 |  |
| **High** | 24 | 67 |  | 27 | 61 |  | - | - |  | 71 | 17 |  |
